# Rational Construction of Indium-TiO_2_ Interfacial Composites for Boosting Urea Electrosynthesis

**DOI:** 10.3390/ma19204299

**Published:** 2026-10-12

**Authors:** Sichao Fu, Zhiwei Wang, Youcai Meng, Mingying Chen, Miaosen Yang, Yang Luo, Sung-Hwan Bae, Xijun Liu

**Affiliations:** 1 School of Chemical Engineering & the Innovation Laboratory for the Manufacturing and Application of New Carbon Materials, Jilin Vocational College of Industry and Technology, Jilin 132000, China; 2 Department of New Material Engineering, Kyungnam University, Changwon-si 51767, Gyeongnam-do, Republic of Korea; 3 MOE Key Laboratory of New Processing Technology for Nonferrous Metals and Materials, School of Resources, Environment and Materials, Guangxi University, Nanning 530004, China; 4 School of Chemical Engineering, Northeast Electric Power University, Jilin 132012, China; 5 Department of Physics, City University of Hong Kong, Hong Kong SAR 999077, China; 6 China Huadian Corporation Ltd. (CHD), Beijing 100031, China

**Keywords:** In–TiO_2_ interfacial composite, electrocatalytic urea synthesis, C–N coupling, density functional theory

## Abstract

The electrocatalytic co-reduction of CO_2_ and nitrate to urea offers a sustainable route for carbon fixation, nitrogen waste recycling, and high-value chemical production. However, the rational design of efficient electrocatalysts with synergistic activation capability toward both reactants and the ability to suppress side reactions remains challenging. Herein, an N-doped carbon-modified In–TiO_2_ interfacial composite catalyst (In–TiO_2_/NC) was fabricated via a solvothermal method followed by high-temperature calcination. The catalyst exhibits a urea yield rate of 618.9 μg·h^−1^·mg_cat._^−1^ at −1.0 V vs. RHE. Control experiments verify that urea is generated through C–N coupling between intermediates derived from CO_2_ reduction and nitrate reduction. Density functional theory calculations indicate that the coupled In and TiO_2_ components strengthen CO_2_ adsorption, maintain favorable nitrate activation, improve the electronic states available for charge transfer, and suppress the hydrogen evolution reaction. This work demonstrates that rational interfacial coupling of catalytic components can promote electrocatalytic urea synthesis from CO_2_ and nitrate, providing a promising strategy for high-performance C–N coupling electrocatalysts.

## 1. Introduction

The rapid expansion of industrial activities has substantially accelerated socioeconomic development, but has also intensified energy consumption and environmental pollution. In particular, the continued dependence on fossil fuels has led to massive CO_2_ emissions and the release of nitrogen oxides (NO_x_), contributing to global warming and environmental deterioration. NO_x_ can be further transformed into nitrate species through atmospheric and aqueous processes, posing potential risks to aquatic ecosystems and groundwater quality [1,2]. Against this background, the development of renewable-energy-driven and environmentally benign chemical processes has become increasingly important for mitigating greenhouse-gas emissions and advancing sustainable development [3,4,5]. Urea, one of the most important nitrogen fertilizers and industrial chemicals, is conventionally produced through the energy-intensive Haber–Bosch process for NH_3_ synthesis followed by the Bosch–Meiser process for urea production. These processes require harsh operating conditions and are associated with substantial energy consumption and carbon emissions, highlighting the urgent need for more sustainable urea synthesis technologies [6,7,8].

Electrocatalytic co-reduction of CO_2_ and nitrate (NO_3_^−^) to urea offers an attractive alternative because it can operate under ambient conditions and be powered by renewable electricity. More importantly, this strategy integrates CO_2_ fixation, nitrate remediation, and value-added urea synthesis through direct electrocatalytic C–N coupling, providing a promising route toward sustainable carbon and nitrogen utilization [9,10,11,12,13]. Nevertheless, the overall reaction involves complex multistep proton-coupled electron-transfer processes and remains limited by sluggish reactant activation, inefficient formation of C–N bonds, and competing side reactions, particularly the hydrogen evolution reaction (HER), which collectively lead to unsatisfactory urea yield and selectivity. Therefore, rationally designing electrocatalysts capable of simultaneously activating CO_2_ and NO_3_^−^, regulating the adsorption and evolution of key carbon- and nitrogen-containing intermediates, and promoting selective C–N coupling is crucial for achieving efficient electrosynthetic urea production [14,15,16].

Titanium dioxide (TiO_2_) is a widely used n-type semiconductor material with excellent chemical stability, controllable crystal structure and surface morphology, and abundant surface defects, including oxygen-deficient sites and reduced Ti species [17,18]. In electrocatalysis, TiO_2_ is often used as a stable support and interface-regulation platform, which can enhance the adsorption and activation of NO_3_^−^ through surface defects and regulate the adsorption behavior of reaction intermediates [19,20]. Recent studies have shown that TiO_2_-based architectures can regulate the reactivity of coupled metal species through interfacial shielding and molecular confinement. For example, molecular shielding in TiO_2_ nanotubes was reported to activate oxovanadium (IV) species for ultrafast aqueous polymerization, highlighting the importance of the confined interfacial environment in controlling metal-centered reactivity [21]. These findings suggest that TiO_2_ can serve not only as a structural support but also as an interface-regulation platform. However, pure TiO_2_ has low intrinsic conductivity and weak CO_2_ activation ability, making it difficult to achieve efficient C–N coupling [22,23,24,25]. Indium (In) exhibits excellent performance in CO_2_ electroreduction and can efficiently activate CO_2_ to produce key intermediates facilitating C–N coupling. Therefore, constructing an in–TiO_2_ interface may combine the CO_2_ activation capability of In with the interfacial regulation and nitrate-affinity characteristics of TiO_2_, although the specific interfacial mechanism requires further investigation. Coupling metallic In with TiO_2_ in a carbon-supported composite is therefore expected to integrate the complementary functions of both components, enabling the synergistic activation of CO_2_ and NO_3_^−^, enhancing C–N coupling efficiency, and suppressing side reactions [26,27,28,29,30].

Based on the above considerations, an N-doped carbon-supported In–TiO_2_ composite (In–TiO_2_/NC) was prepared using a solvothermal method followed by high-temperature calcination. The morphology, crystal structure, and surface chemistry of In–TiO_2_/NC were systematically characterized, and its electrocatalytic performance in converting CO_2_ and nitrates to urea was evaluated. By combining control experiments and density functional theory (DFT) calculations, the study investigated the roles of the In and TiO_2_ components in reactant adsorption, electronic state modulation, and the suppression of competing reactions. This research provides a viable strategy for designing highly efficient electrocatalysts for urea synthesis and offers new insights into the coupled utilization of carbon dioxide and nitrates.

## 2. Materials and Methods

### 2.1. Catalyst Synthesis

#### 2.1.1. Preparation of In–TiO_2_/NC

30 mL of N,N-dimethylformamide (DMF, Aladdin, Shanghai, China) and 5 mL of methanol(Sinopharm Chemical Reagent Co., Ltd., Shanghai, China) were mixed together. 3 mmol of 2-NH_2_BDC (Aladdin, Shanghai, China), 3 mmol of In(NO_3_)_3_·xH_2_O (Aladdin, Shanghai, China), and 1 mmol of TiO_2_ nanoparticles(Aladdin, Shanghai, China) were added into the above solution, corresponding to a nominal molar ratio of 2-NH_2_BDC: In precursor: TiO_2_ = 3:3:1. The mixture was ultrasonicated for 30 min and then stirred for another 30 min to ensure the solid particles were fully dispersed. Subsequently, the solution was transferred into a 100 mL Teflon-lined stainless-steel autoclave and heated at 160 °C for 12 h. After cooling to room temperature, the product was washed with DMF and ethanol (Sinopharm Chemical Reagent Co., Ltd., Shanghai, China) alternately at least three times, and dried in a vacuum oven at 60 °C overnight. Finally, the sample was heated to 900 °C at a heating rate of 5 °C min^−1^ and kept for 2 h. The obtained sample was denoted as In–TiO_2_/NC. Samples with different TiO_2_ contents were prepared by adjusting the addition ratio, denoted as In–TiO_2(0.3)_/NC, In–TiO_2(1)_/NC, In–TiO_2(3)_/NC, and In–TiO_2(9)_/NC.

#### 2.1.2. Preparation of In/NC

In/NC was synthesized by the same procedure as In–TiO_2_/NC, except that only 3 mmol of 2-NH_2_BDC and 3 mmol of In(NO_3_)_3_·xH_2_O were added to the solution without TiO_2_.

#### 2.1.3. Preparation of TiO_2_/NC

TiO_2_/NC was synthesized by the same procedure as In–TiO_2_/NC, except that only 3 mmol of 2-NH_2_BDC and 1 mmol of TiO_2_ were added to the solution without an indium source.

#### 2.1.4. Preparation of M–TiO_2_/NC

In previous exploratory experiments, transition metals, rare-earth metals, and noble metals were used to regulate and construct heterostructures for urea synthesis. Here, M represents metals including Cu, Co, Fe, Zn, In, V, Y, Pd, and Ce. M–TiO_2_/NC was synthesized by the same procedure as In–TiO_2_/NC, with a molar ratio of organic ligand: metal salt: TiO_2_ = 1:1:1.

### 2.2. Electrochemical Test

All electrochemical tests were performed on a CHI 760F electrochemical workstation using a well-sealed H-type cell with two chambers separated by a Nafion-117 proton exchange membrane. Ag/AgCl electrode and a graphite rod were used as the reference electrode and counter electrode, respectively. Both cathodic and anodic chambers were filled with 0.1 M KHCO_3_ + 50 mM KNO_3_ electrolyte. The cathodic chamber was mechanically stirred to enhance mass transfer and reduce concentration polarization.

Pretreatment of Nafion-117 membrane. The Nafion-117 membrane was pretreated before use. The membrane was boiled in 5 wt% H_2_O_2_ solution at 80 °C for 1 h, washed in deionized water at 80 °C for 1 h, boiled in 0.05 M H_2_SO_4_ solution at 80 °C for 1 h, and washed thoroughly in deionized water at 80 °C for 3 h. The treated membrane was stored in deionized water before use.

Preparation of working electrode. 2 mg of catalyst powder was dispersed into a mixture of 950 μL ethanol and 50 μL Nafion solution (5 wt%). The mixture was ultrasonicated for 30 min to form a homogeneous catalyst ink. 50 μL of the ink was dropped onto a hydrophilic carbon paper (1 × 1 cm^2^) and dried under an infrared lamp. The final catalyst loading was controlled at 0.1 mg·cm^−2^.

Electrochemical measurements. Before tests, CO_2_ was purged into the cathodic chamber at a flow rate of 50 mL·min^−1^ for 30 min to saturate the electrolyte. During tests, the CO_2_ flow rate was maintained at 30 mL·min^−1^ to avoid disturbance. All potentials were converted to the reversible hydrogen electrode (RHE) scale using the following equation: E (vs. RHE) = E (vs. Ag/AgCl) + 0.0591 × pH + 0.197.

Cyclic Voltammetry (CV) tests were conducted in the potential range of 0 to −1.4 V vs. RHE at a scan rate of 0.1 V·s^−1^ for 30 cycles until stable curves were obtained.

Linear Sweep Voltammetry (LSV) tests were performed from 0 to −1.4 V vs. RHE at a scan rate of 10 mV·s^−1^ to evaluate the catalytic activity toward CO_2_ reduction, NO_3_^−^ reduction, and their coupling reactions.

Amperometric i–t Curve. Constant-potential electrolysis for potential-dependent activity measurements was carried out at various applied potentials for 30 min at each potential. The current–time curves were recorded continuously. Products were sampled for concentration analysis, and the yield and Faradaic efficiency were calculated. The calibration curves used for the quantification of urea, NH_3_/NH_4_^+^, and NO_2_^−^ are provided in Figures S3, S4, and S5, respectively. For the long-term stability test, chronoamperometry was conducted at −1.0 V vs. RHE for 30 h.

The double-layer capacitance (*C_dl_*) method was used to evaluate the electrochemically active surface area (ECSA). CV curves were recorded at scan rates of 50, 60, 70, 80, 90, and 100 mV s^−1^. At a selected non-Faradaic potential, the difference between the anodic and cathodic current densities (Δ*j* = *j_a_* − *j_c_*) was plotted against the scan rate (*v*). The *C_dl_* value was calculated according to:

∆*j* = 2*C_dl_v*

where *j_a_* and *j_c_* are the anodic and cathodic current densities, respectively, and *v* is the scan rate. The ECSA was related to *C_dl_* according to:

$$\mathrm{ECSA}=\frac{C_{\mathrm{dl}}}{C_{s}},$$

where *C_s_* is the specific capacitance of the electrode surface [10].

Electrochemical Impedance Spectroscopy (EIS) measurements were conducted at the open-circuit potential (OCP) in a frequency range of 0.01 Hz to 10^5^ Hz with an AC amplitude of 5 mV. The data were fitted using ZView3.0 (Scribner Associates Inc., USA, 2009) software to obtain the solution resistance (R_s_) and charge-transfer resistance (R_ct_).

### 2.3. Density Functional Theory (DFT) Calculations

The crystallographic information file (CIF) of TiO_2_ was obtained from the Inorganic Crystal Structure Database (ICSD), and the computational model was constructed using the Hongzhiwei computing platform. DFT calculations were performed using DS-PAW, a first-principles calculation software package in Hongzhiwei Device Studio 2025B (Hongzhiwei Technology (Shanghai) Co., Ltd., Shanghai, China, 2025). The generalized gradient approximation (GGA) with the Perdew–Burke–Ernzerhof (PBE) functional was employed to describe the exchange-correlation interactions.

Structural relaxations were performed using a 2 × 2 × 1 Gamma-centered k-point mesh with a kinetic energy cutoff of 450 eV. The convergence criteria for structural relaxation and energy calculations were set to a maximum atomic force of 0.03 eV·Å^−1^. A 15 Å vacuum layer was introduced along the z-axis of the TiO_2_ slab to avoid spurious interactions between periodic images. The density of states (DOS) was calculated using the same parameters to analyze electronic interactions during the reaction. The optimized structural models of TiO_2_/NC, In/NC, and In–TiO_2_/NC, together with the corresponding CO_2_ and NO_3_^−^ adsorption configurations, are provided in Figure S1 of the Supporting Information.

The adsorption energies of key intermediates for urea synthesis ($E_{\left(CO_{2}\right)}$/$E_{\left(NO_{3}^{-}\right)}$) were calculated as:

$$E_{\left(CO_{2}\right)}=E_{\left(CO_{2}+\mathrm{slab}\right)}-E_{\left(CO_{2}\right)}-E_{\left(\mathrm{slab}\right)}$$

$$\mathrm{br}-\mathrm{to}-\mathrm{break}\;E_{\left(NO_{3}^{-}\right)}=E_{\left(NO_{3}^{-}+\mathrm{slab}\right)}-E_{\left(NO_{3}^{-}\right)}-E_{\left(\mathrm{slab}\right)}$$

where $E_{\left(CO_{2}+\mathrm{slab}\right)}$ and $E_{\left(NO_{3}^{-}+\mathrm{slab}\right)}$ are the total energies of CO_2_ or NO_3_^−^ adsorbed on the slab, $E_{\left(CO_{2}\right)}$ and $E_{\left(NO_{3}^{-}\right)}$ are the energies of isolated CO_2_ and NO_3_^−^, and *E*_(slab)_ is the energy of the clean slab.

## 3. Results and Discussion

### 3.1. Characterization of the Catalysts

**Figure 1:**
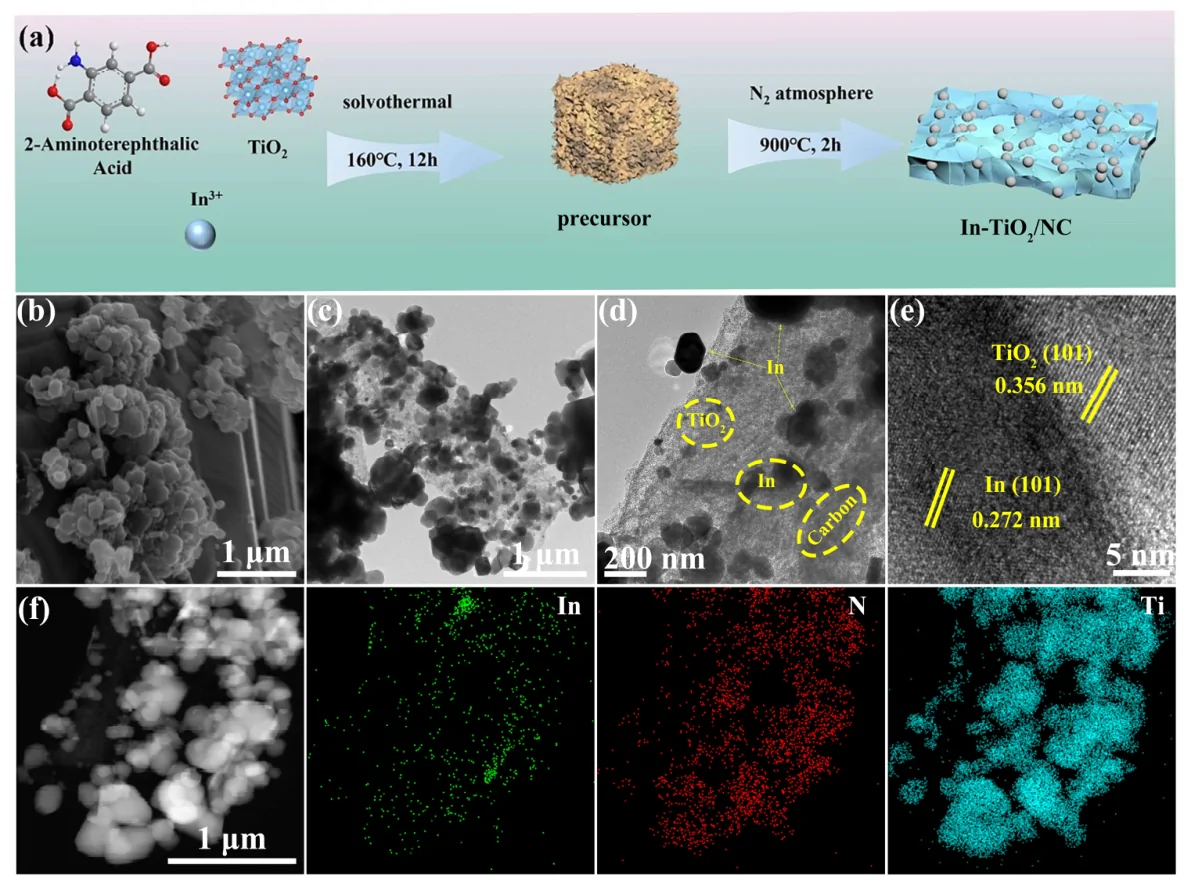
(**a**) Schematic illustration of the catalyst synthesis. (**b**) SEM image of In–TiO_2_/NC. (**c**,**d**) TEM images of In–TiO_2_/NC. (**e**) HRTEM image of In–TiO_2_/NC. (**f**) HAADF-STEM image and corresponding elemental mappings of In, N, and Ti, respectively.

Figure 1a schematically illustrates the preparation of In–TiO_2_/NC through a solvothermal process followed by high-temperature calcination under an N_2_ atmosphere. Specifically, the In precursor, TiO_2_ nanoparticles, and 2-NH_2_BDC ligand were first assembled in a DMF/methanol solvent, followed by washing and drying. The resulting precursor was then calcined at 900 °C under N_2_ to form the N-doped carbon-supported In–TiO_2_ composite. The morphology and microstructure of the catalyst were examined by scanning electron microscopy (SEM) and transmission electron microscopy (TEM). As shown in Figure 1b,c, In–TiO_2_/NC exhibits an aggregated particulate morphology consisting of relatively large domains and smaller nanoparticles. Additional TEM images of the reference samples, In/NC and TiO_2_/NC, are provided in Figure S2 for morphological comparison. In/NC exhibits relatively large high-contrast domains, whereas TiO_2_/NC is mainly composed of aggregated TiO_2_ nanoparticles with characteristic sizes of several tens of nanometers. A continuous carbon coating layer can be clearly observed on the sample surface in Figure 1d. The formation of this carbon layer suggests that the organic precursor undergoes carbonization during heat treatment and is finally retained on the catalyst surface. The introduction of the carbon layer not only helps to inhibit further sintering and structural evolution of particles at high temperatures but also improves the electrical conductivity of the system, which is beneficial to electron transfer during the electrocatalytic process [31,32]. The local crystalline structure of In–TiO_2_/NC was further examined by HRTEM. As shown in Figure 1e, lattice spacings of approximately 0.272 and 0.356 nm are observed, corresponding to the (101) plane of metallic In and the (101) plane of anatase TiO_2_, respectively. These results confirm the nanoscale coexistence of crystalline In and TiO_2_ in the composite. The HAADF-STEM image and corresponding elemental mappings are shown in Figure 1f. The elements In, Ti, and N are uniformly distributed throughout the catalyst particle, demonstrating that all components are well dispersed and combined during the preparation process. This result further validates the stability and uniformity of the as-constructed In–TiO_2_/NC structure, laying a reliable structural foundation for the subsequent study of catalytic performance. Additionally, the In loading amount is measured to be 7.26 wt% by Inductively Coupled Plasma (ICP).

**Figure 2:**
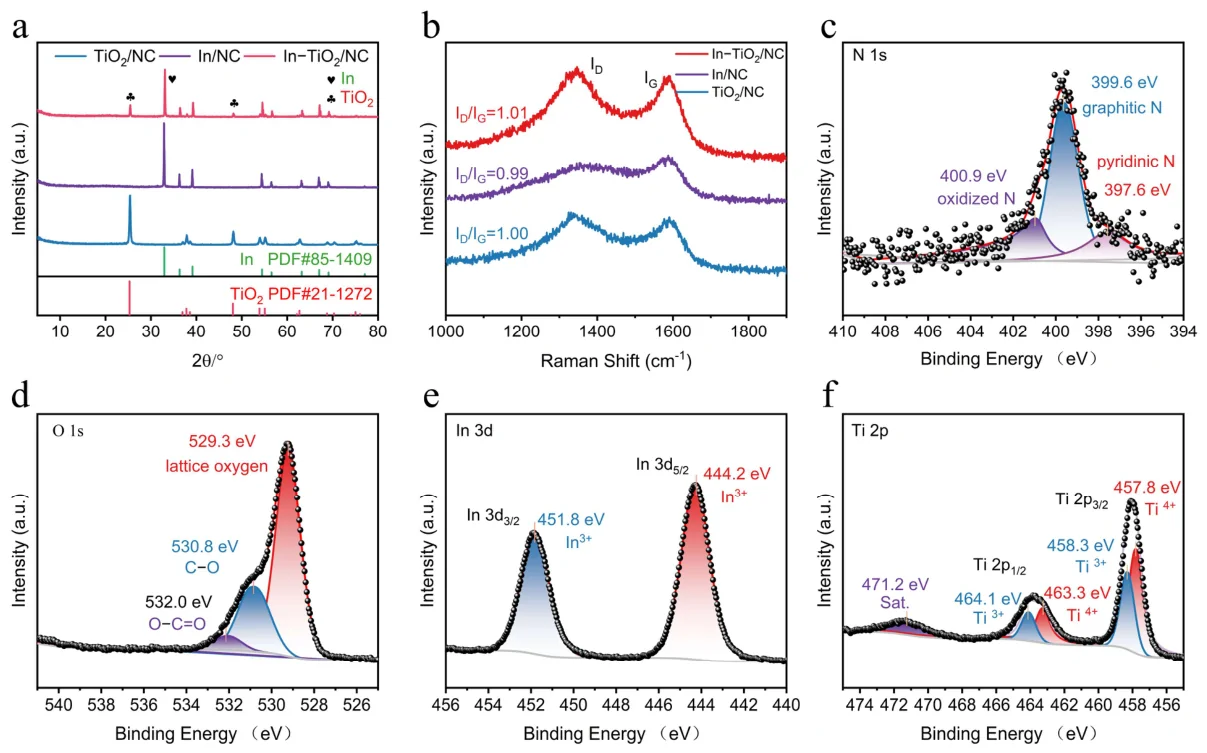
(**a**) XRD patterns of In–TiO_2_/NC, In/NC, and TiO_2_/NC. (**b**) Raman spectra of In–TiO_2_/NC, In/NC, and TiO_2_/NC. High-resolution XPS spectra for (**c**) N 1s, (**d**) O 1s, (**e**) In 3d, and (**f**) Ti 2p of In–TiO_2_/NC.

The phase composition and crystal structure of the as-prepared In–TiO_2_/NC, In/NC, and TiO_2_/NC samples were characterized by X-ray diffraction (XRD). As shown in Figure 2a, the diffraction pattern of In–TiO_2_/NC matches well with those of metallic In (PDF#85-1409) and anatase TiO_2_ (PDF#21-1272), confirming the coexistence of the two crystalline phases. The characteristic diffraction peak at 32.9° is assigned to the (101) plane of metallic In, while the peaks at 25.3° and 38.0° correspond to the (101) and (200) planes of anatase TiO_2_, respectively. In comparison, In/NC predominantly exhibits the diffraction features of metallic In, whereas TiO_2_/NC shows the characteristic reflections of anatase TiO_2_, further supporting the coexistence of In and TiO_2_ in the In–TiO_2_/NC composite [18]. No additional diffraction peaks attributable to crystalline In nitride or other detectable impurity phases are observed, indicating that In is predominantly present as a metallic crystalline phase after high-temperature calcination. Raman spectroscopy was employed to evaluate the defect degree of the carbon component (Figure 2b). The peaks at approximately 1350 cm^−1^ and 1580–1600 cm^−1^ belong to the D band (defective carbon) and G band (graphitic sp^2^ carbon), respectively. The I_D_/I_G_ ratios of In–TiO_2_/NC, In/NC, and TiO_2_/NC are 1.01, 0.99, and 1.00, respectively. The comparable I_D_/I_G_ ratios indicate similar defect characteristics among the carbon matrices, while the slightly higher ratio of In–TiO_2_/NC suggests a modestly greater contribution from defect-related carbon features. These defect-related features may facilitate reactant adsorption and electrocatalytic reactions [33,34].

X-ray photoelectron spectroscopy (XPS) was further conducted to characterize the surface chemical states and elemental composition (Figure 2c–f). The N 1s spectrum can be deconvoluted into pyridinic N (397.6 eV), graphitic N (399.6 eV), and oxidized N (400.9 eV), indicating that N atoms have been successfully doped into the carbon framework, which can regulate the local electronic structure and enhance the electrocatalytic activity [35]. The O 1s spectrum confirms the existence of lattice oxygen in TiO_2_, C–O, and O–C=O groups [19]. In the Ti 2p spectrum, typical peaks of Ti^4+^ and Ti^3+^ are observed, demonstrating the existence of oxygen vacancies and Ti^3+^ defects, which are beneficial to the adsorption and activation of NO_3_^−^ [30]. The In 3d spectrum shows characteristic peaks of metallic In, further confirming the successful introduction of In species. The above results jointly demonstrate that the In–TiO_2_/NC interfacial composite with Ti^3+^-associated oxygen-deficient surface environments, N-doped carbon layer, uniform elemental distribution, and strong electronic interaction has been successfully fabricated.

### 3.2. Electrocatalytic Performance

**Figure 3:**
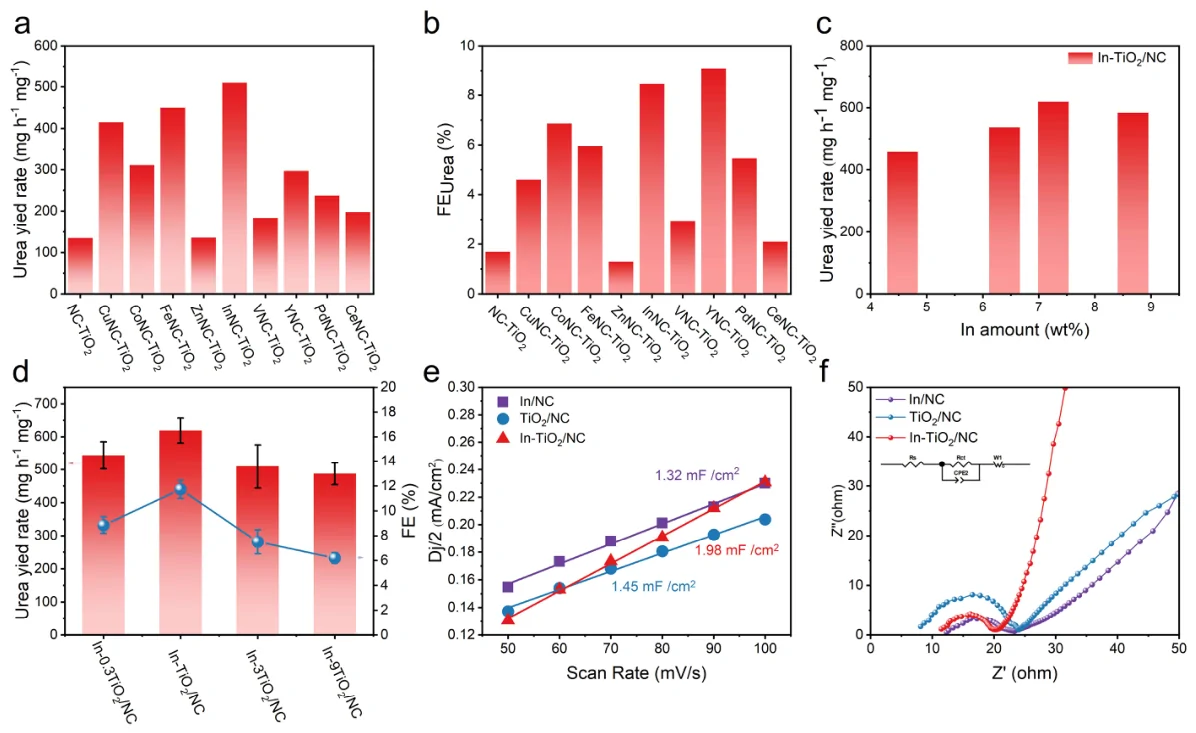
(**a**) Urea yield rates and (**b**) Faradaic efficiencies of M–TiO_2_/NC catalysts with different metal components. (**c**) Urea yield rates of In–TiO_2_/NC with different In contents. (**d**) Urea yield rates and corresponding Faradaic efficiencies of In–TiO_2_/NC prepared with different TiO_2_ amounts. (**e**) C_dl_ analysis of In/NC, TiO_2_/NC, and In–TiO_2_/NC. (**f**) EIS Nyquist plots and equivalent-circuit fitting results of the three catalysts.

The metal component was first screened using a series of M–TiO_2_/NC catalysts. As shown in Figure 3a,b, In–TiO_2_/NC exhibited the highest urea yield rate together with a relatively high Faradaic efficiency, and was therefore selected for further investigation. In–TiO_2_/NC exhibits the highest urea yield rate among the investigated samples. Although Y–TiO_2_/NC shows a relatively high Faradaic efficiency (FE), its urea yield rate is lower than that of In–TiO_2_/NC. In addition, the optimal In loading amount is found to be 7.26 wt% as revealed by Figure 3c. Considering both urea yield and FE, In–TiO_2_/NC with 7.26 wt% was selected for further investigation. The TiO_2_ content was subsequently optimized. As shown in Figure 3d and Figure S6a, the urea production first increases and then decreases with increasing TiO_2_ dosage, with the highest performance obtained at an organic ligand: In precursor, TiO_2_ molar ratio of 3:3:1. In contrast, ammonia (NH_3_) production increases with increasing TiO_2_ content, indicating increased competition from nitrate reduction toward NH_3_ at higher TiO_2_ loadings. The corresponding XRD patterns are shown in Figure S6b.

**Figure 4:**
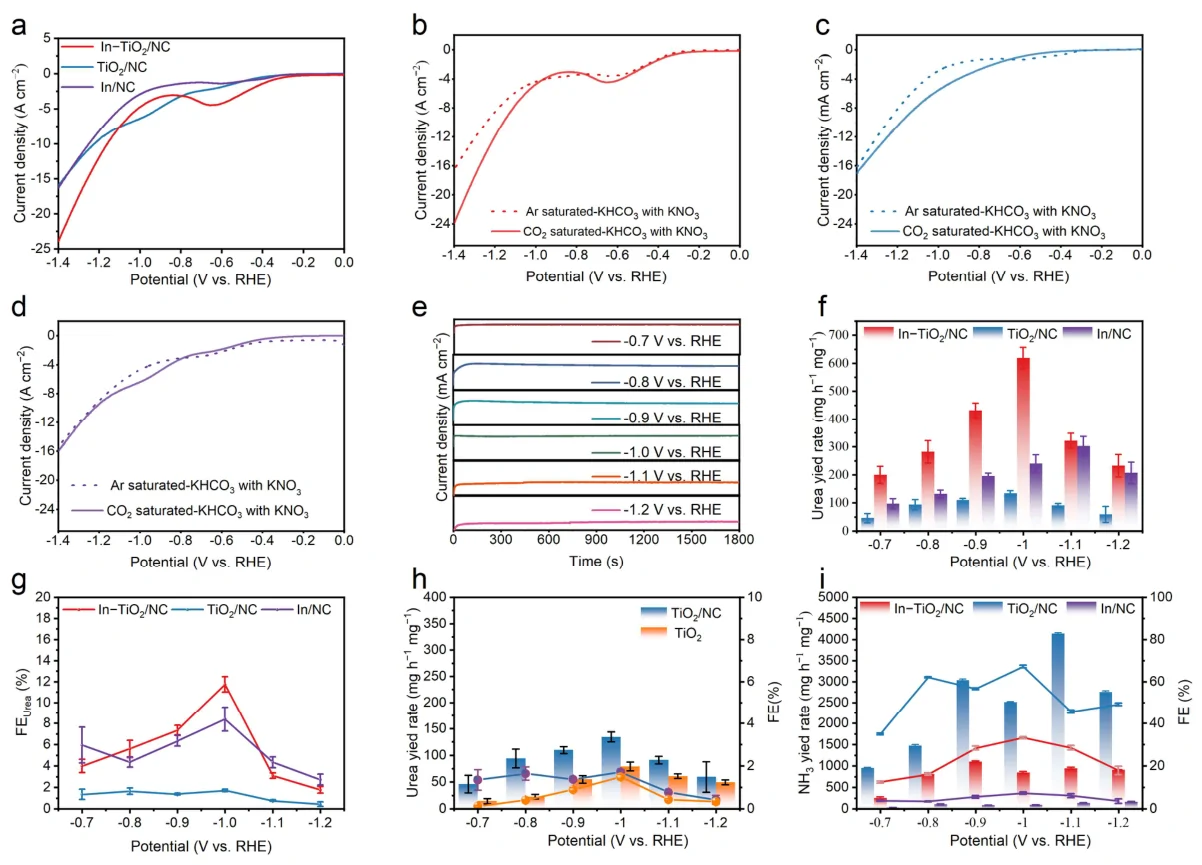
(**a**) LSV curves of In−TiO_2_/NC, In/NC, and TiO_2_/NC. (**b**–**d**) LSV curves of the three catalysts recorded under Ar- and CO_2_-saturated conditions. (**e**) Chronoamperometric curves of In−TiO_2_/NC recorded at different applied potentials. (**f**) Urea yield rates and (**g**) corresponding Faradaic efficiencies of the three catalysts at different potentials. (**h**) FE distribution of the quantified products over In−TiO_2_/NC at different applied potentials. (**i**) NH_3_ yield rates and corresponding Faradaic efficiencies of In−TiO_2_/NC, In/NC, and TiO_2_/NC at different potentials.

To evaluate the electrocatalytic performance of the synthesized catalysts, a series of electrochemical measurements were conducted using a conventional three-electrode system in an H-battery configuration. Electrochemical double-layer capacitance (C_dl_) measurements were first performed to compare the electrochemically accessible surface areas of the catalysts. As shown in Figure 3e and Figure S7, In–TiO_2_/NC exhibits a C_dl_ value of 1.98 mF cm^−2^, higher than those of In/NC (1.32 mF cm^−2^) and TiO_2_/NC (1.45 mF cm^−2^), suggesting a larger electrochemically accessible surface area. Electrochemical impedance spectroscopy (EIS) was further used to compare their charge-transfer behavior. As shown in Figure 3f, In–TiO_2_/NC exhibits a smaller high-frequency semicircle than In/NC and TiO_2_/NC, corresponding to a lower charge-transfer resistance at the electrode/electrolyte interface. Collectively, the outstanding activity of In–TiO_2_/NC arises from the synergistic combination of its large ECSA and rapid charge-transport capability. As shown in Figure 4a, in linear sweep voltammetry (LSV), In–TiO_2_/NC exhibits a higher cathodic current density than In/NC and TiO_2_/NC under the CO_2_/NO_3_^−^ co-reduction conditions. Detailed LSV curves recorded under different gas atmospheres are provided in Figure 4b–d. The current responses change after CO_2_ introduction, with In–TiO_2_/NC showing the most pronounced response among the three catalysts. The corresponding chronoamperometric curves recorded at different applied potentials are provided in Figure 4e. Potentiostatic electrolysis was subsequently conducted in CO_2_-saturated electrolyte to evaluate urea production at different potentials. As shown in Figure 4f,g, both the urea yield rate and FE first increase and then decrease as the applied potential becomes more negative. In–TiO_2_/NC achieves the highest performance at −1.0 V vs. RHE, with a urea yield rate of 618.9 μg h^−1^ mg_cat_^−1^ and an FE of 11.7%, compared with 239.7 μg h^−1^ mg_cat_^−1^ and 8.4% for In/NC and 134.5 μg h^−1^ mg_cat_^−1^ and 1.7% for TiO_2_/NC, respectively. A comparison of the urea yield rate and Faradaic efficiency of In–TiO_2_/NC with those of recently reported catalysts is summarized in Table S1. At potentials more negative than −1.0 V vs. RHE, both the urea yield rate and FE decrease, indicating increased competition from other electroreduction pathways. The contribution of the N-doped carbon component was further evaluated by comparing TiO_2_/NC with pure TiO_2_. As shown in Figure 4h and Figure S8a, TiO_2_/NC exhibits higher urea and NH_3_ yields than pure TiO_2_ over the investigated potential range. At −1.0 V vs. RHE, the urea yield rate of TiO_2_/NC reaches 134.5 μg h^−1^ mg_cat_^−1^, compared with 94.7 μg h^−1^ mg_cat_^−1^ for pure TiO_2_. The corresponding XRD patterns (Figure S8b) show that TiO_2_/NC retains the anatase phase after high-temperature treatment, supporting the role of the carbon component in maintaining the TiO_2_ crystalline phase.

**Figure 5:**
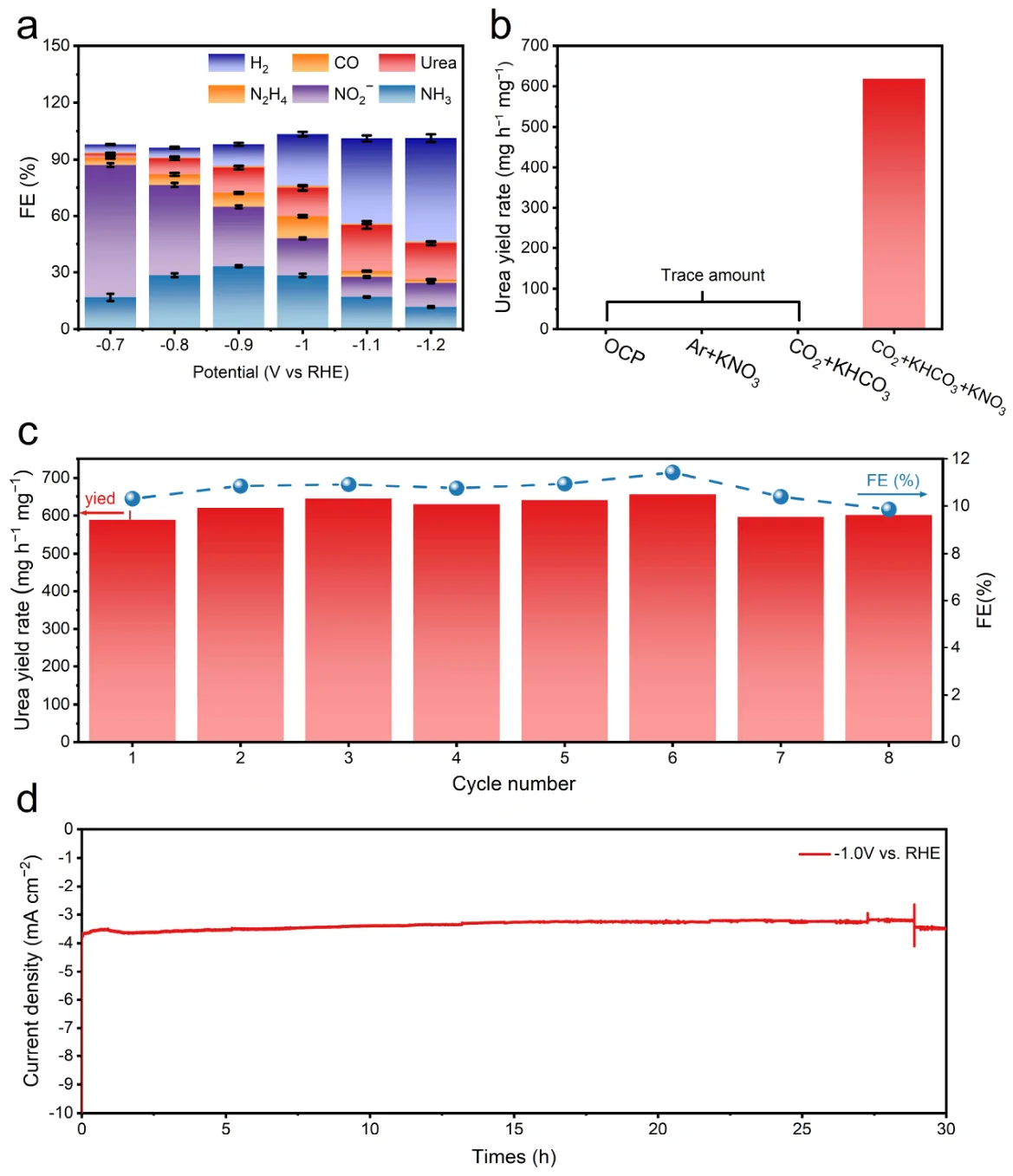
(**a**) FE distribution of the quantified products over In−TiO_2_/NC at different applied potentials. (**b**) Urea yield rate of In−TiO_2_/NC under different control conditions. (**c**) Cycling stability of In−TiO_2_/NC over eight consecutive cycles at −1.0 V vs. RHE. (**d**) Long−term chronoamperometric stability of In−TiO_2_/NC at −1.0 V vs. RHE for 30 h.

NH_3_ formation was evaluated to examine the competition between nitrate reduction and urea synthesis. As shown in Figure 4i, TiO_2_/NC exhibits the highest NH_3_ FE, reaching approximately 67.2%, whereas the corresponding values for In–TiO_2_/NC and In/NC are approximately 28.4% and 7.1%, respectively. These results indicate a stronger tendency of TiO_2_/NC toward nitrate reduction to NH_3_, while incorporation of the In-containing component changes the product distribution under the co-reduction conditions. Recent studies have shown that regulating the hydrogenation pathways of nitrogen-containing intermediates is important for improving the selectivity of urea electrosynthesis [36]. The product distribution of In–TiO_2_/NC at different applied potentials is shown in Figure 5a. The quantified products include NH_3_, NO_2_^−^, N_2_H_4_, H_2_, CO, and urea. The urea FE reaches its maximum at −1.0 V vs. RHE and decreases at more negative potentials [37,38]. Control experiments were performed under different reaction conditions. As shown in Figure 5b, only trace amounts of urea are detected under open-circuit conditions and in the incomplete-reactant control systems, whereas a substantially higher urea yield is obtained in the CO_2_/KHCO_3_/KNO_3_ system. Component-specific blank tests were further conducted to evaluate possible background contributions from the electrolyte, membrane, carbon paper, and KNO_3_ reagent (Figure S9). In the ^15^NO_3_^−^−labeling experiment, the ^15^N NMR signal at approximately 78 ppm was assigned to the amide nitrogen of ^15^N-labeled urea (Figure S10a). In the ^13^CO_2_-labeling experiment, the ^13^C NMR signal at approximately 163 ppm was assigned to the carbonyl carbon of ^13^C-labeled urea (Figure S10b). These results support the incorporation of nitrate-derived nitrogen and CO_2_-derived carbon into urea, providing evidence for the proposed C–N coupling pathway [39]. The operational stability of In–TiO_2_/NC was evaluated at −1.0 V vs. RHE. As shown in Figure 5c, the urea yield rate and FE remain comparable over eight consecutive electrolysis cycles. The 30 h chronoamperometric measurement also shows a relatively stable current response (Figure 5d). The XRD pattern collected after the 30 h chronoamperometric stability test is provided in Figure S11. To further evaluate the catalyst after the 30 h chronoamperometric stability test, TEM characterization and elemental-ratio analysis were performed. The catalyst morphology and elemental composition remained essentially unchanged (Figure S12 and Table S2), further supporting the operational stability of In–TiO_2_/NC under the investigated conditions. These results demonstrate satisfactory operational stability of In–TiO_2_/NC.

### 3.3. Mechanism and Theoretical Calculations

**Figure 6:**
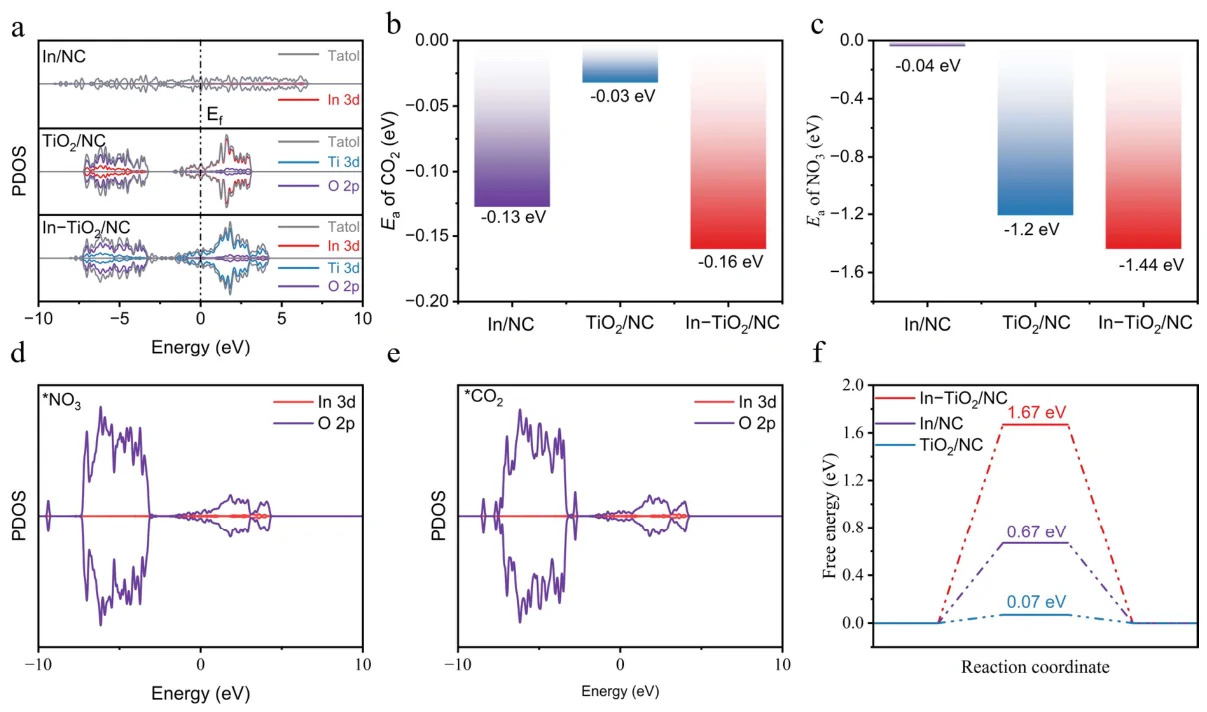
(**a**) PDOS of In/NC, TiO_2_/NC, and In–TiO_2_/NC, with the Fermi level set to 0 eV. Calculated adsorption energies of (**b**) CO_2_ and (**c**) NO_3_^−^ on In/NC, TiO_2_/NC, and In–TiO_2_/NC. Orbital-resolved density of states for (**d**) *NO_3_^−^ and (**e**) *CO_2_ -adsorbed configurations on In–TiO_2_/NC. (**f**) Calculated free-energy profiles for H^*^ adsorption on In/NC, TiO_2_/NC, and In–TiO_2_/NC.

To clarify the origin of the catalytic behavior, density functional theory calculations were carried out to analyze the electronic structures, reactant adsorption, and H^*^ adsorption of In/NC, TiO_2_/NC, and In–TiO_2_/NC (Figure 6). The PDOS results (Figure 6a) show clear differences in the electronic structures of the three models. In/NC exhibits continuous electronic states near the Fermi level, whereas TiO_2_/NC shows a much lower density of states around the Fermi level. In comparison, In–TiO_2_/NC presents a more continuous state distribution near the Fermi level than TiO_2_/NC, indicating a modified electronic structure after coupling the In- and TiO_2_-containing components [40,41,42].

As shown in Figure 6b, the adsorption energies of CO_2_ on In/NC, TiO_2_/NC, and In–TiO_2_/NC are −0.13, −0.03, and −0.16 eV, respectively, indicating that In–TiO_2_/NC provides the most favorable interaction with CO_2_ among the three models. For NO_3_^−^ adsorption (Figure 6c), the corresponding values are −0.04, −1.20, and −1.44 eV. This result shows that TiO_2_/NC has a much stronger affinity for NO_3_^−^ than In/NC, while In–TiO_2_/NC further strengthens NO_3_^−^ adsorption. Taken together, these results suggest that In–TiO_2_/NC combines favorable adsorption toward both CO_2_ and NO_3_^−^ [43,44]. The adsorption-state PDOS analyses provide additional insight into the reactant–surface interaction. For both *NO_3_ and *CO_2_ adsorbed on In–TiO_2_/NC (Figure 6d,e), the O 2p states of the adsorbates overlap with the electronic states of the catalyst, indicating electronic interaction upon adsorption. These features are consistent with the calculated adsorption behavior and support the effective interaction of both reactants with the In–TiO_2_/NC surface [45,46].

The thermodynamic tendency toward the competing hydrogen evolution reaction was evaluated using the free energy of H^*^ adsorption (Figure 6f). TiO_2_/NC shows the smallest ΔG_H_^*^ value (0.07 eV), indicating more favorable H^*^ adsorption, whereas the values for In/NC and In–TiO_2_/NC are 0.67 and 1.67 eV, respectively. The much larger ΔG_H_^*^ of In–TiO_2_/NC indicates markedly weakened H^*^ adsorption, implying reduced HER tendency on the composite surface [47]. Therefore, the calculated results suggest that In–TiO_2_/NC not only provides favorable adsorption toward both CO_2_ and NO_3_^−^, but also suppresses the competing hydrogen evolution reaction, which is beneficial for the co-reduction process.

## 4. Conclusions

In summary, a novel N-doped carbon-modified In–TiO_2_ interfacial composite catalyst (In–TiO_2_/NC) was developed for electrocatalytic urea production from CO_2_ and nitrate. The combination of metallic In, anatase TiO_2_, and N-doped carbon provides close nanoscale contact among complementary components, a conductive carbonaceous matrix, and accessible catalytic sites. The optimized In–TiO_2_/NC catalyst delivers a urea yield rate of 618.9 μg·h^−1^·mg_cat._^−1^ and a Faradaic efficiency of 11.7% at −1.0 V vs. RHE, together with stable electrolysis behavior over 30 h. Control experiments support the participation of CO_2_ and nitrate in the urea-formation process. DFT calculations further indicate that the coupled In and TiO_2_ components provide favorable adsorption toward CO_2_ and nitrate, regulate adsorbate-associated electron redistribution, and suppress HER, thereby creating favorable conditions for C–N coupling. This study highlights multicomponent interfacial coupling as a useful strategy for developing electrocatalysts toward sustainable urea synthesis and the concurrent utilization of carbon- and nitrogen-containing feedstocks.

## Data Availability

The original contributions presented in this study are included in the article/Supplementary Material. Further inquiries can be directed to the corresponding authors.

## Supplementary Materials

The following supporting information can be downloaded at: https://www.mdpi.com/article/10.3390/ma19204299/s1, Figure S1: Optimized slab models of In/NC, TiO_2_/NC, and In–TiO_2_/NC before and after CO_2_ or NO_3_^–^ adsorption; Figure S2: TEM images of (a) In/NC and (b,c) TiO_2_/NC at different magnifications; Figure S3: (a) Absorbance curves of Urea at different concentrations; (b) Standard curve of Urea; Figure S4: (a) Absorbance curves of NH_4_^+^ at different concentrations; (b) Standard curve of NH_4_^+^; Figure S5: (a) Absorbance curves of NO_2_^–^ at different concentrations; (b) Standard curve of NO_2_^–^; Figure S6: (a) Ammonia nitrogen performance of In–TiO_2_/NC with different molar amounts of TiO_2_ introduced and (b) XRD patterns; Figure S7: CV curves of (a) In/NC; (b) TiO_2_/NC; (c) In–TiO_2_/NC recorded at different scan rates; Figure S8: Electrocatalytic performance of TiO_2_/NC and pure TiO_2_ at different potentials for (a) ammonia synthesis; (c) corresponding XRD patterns; Figure S9: Urea concentrations detected in component-specific blank systems containing the electrolyte, membrane, carbon paper, or pure KNO_3_ under the same electrochemical and analytical conditions. No catalyst was added; Figure S10: Isotope-labeling NMR spectra of urea: (a) ^15^N NMR spectrum from the CO_2_/^15^NO_3_^–^ co-reduction experiment; (b) ^13^C NMR spectrum from the ^13^CO_2_/NO_3_^–^ co-reduction experiment; Figure S11: XRD patterns of In–TiO_2_/NC before and after the 30 h stability test at −1.0 V vs. RHE; Figure S12: TEM image of In–TiO_2_/NC after the 30 h chronoamperometric stability test at −1.0 V vs. RHE; Table S1: Comparison of electrocatalytic urea synthesis performance of reported bimetallic and defect-engineered catalysts.; Table S2: The atomic ratios of In–TiO_2_/NC before and after the electrolysis from the EDX results. Refs. [10,29,48,49,50,51,52,53,54] can be found in Supplementary Materials.
