# Supplementary material for: Rational Construction of Indium-TiO2 Interfacial Composites for Boosting Urea Electrosynthesis
Source: Materials (Basel). 2026 Oct 12;19(20):4299. doi: 10.3390/ma19204299 (PMC13707044; doi:10.3390/ma19204299)
Supplement: Supplementary file 1 [file materials-19-04299-s001.zip › materials-4588071-supplementary.pdf]

Supporting Information for

Rational Construction of Indium-TiO_2 Interfacial Composites for Boosting Urea Electrosynthesis

Sichao Fu [1,2,†], Zhiwei Wang [3,†], Youcai Meng [3,†], Mingying Chen [3], Miaosen Yang [4], Yang Luo [5,6,*], Sung-Hwan Bae [2,*] and Xijun Liu [3,*]

[1] Jilin Vocational College of Industry and Technology, Jilin 13200, China
[2] Kyungnam University, Changwon-si 51767, Gyeongnam-do, Republic of Korea
[3] MOE Key Laboratory of New Processing Technology for Nonferrous Metals and Materials, School of Resources, Environment and Materials, Guangxi University, Nanning 530004, China;
[4] School of Chemical Engineering, Northeast Electric Power University, Jilin 132012, China
[5] Department of Physics, City University of Hong Kong, Hong Kong SAR 999077, China
[6] China Huadian Corporation Ltd. (CHD), Beijing 100031, China
* Correspondence: yluo24-c@my.cityu.edu.hk (Y.L.); shbae@kyungnam.ac.kr (S.-H.B.); xjliu@gxu.edu.cn (X.L.)
† These authors contributed equally to this work.

Experimental section

Materials and reagents

All chemical reagents were of analytical grade and used as received without further purification. Indium(III) nitrate hydrate ($In(NO_3)_3 \cdot xH_2O$), TiO_2 nanoparticles, 2-aminoterephthalic acid (2-NH_2BDC), urea, potassium bicarbonate ($KHCO_3$), potassium nitrate (KNO_3), ethanol, and Nafion solution (5 wt%) were purchased from commercial suppliers. Deionized water was used throughout all experiments.

Characterization

X-ray diffraction (XRD) measurement was carried out using a Bruker D8 ADVANCE diffractometer. X-ray photoelectron spectroscopy (XPS) was conducted on a Thermo Fisher Scientific ESCALAB 250Xi+ spectrometer. Raman spectra were recorded on a Renishaw inVia Reflex Raman spectrometer (UK) to characterize the defect features of the catalysts. Scanning electron microscope (SEM) images were obtained using a Zeiss ULTRA-55 field-emission scanning electron microscope. High-resolution transmission electron microscopy (HRTEM) characterization was carried out on a FEI Tecnai G2 F20 field-emission transmission electron microscope. A UV1800 PC spectrometer was used to record the ultraviolet-visible absorption spectra. Nuclear Magnetic Resonance (NMR) qualitative analysis of ammonia (as NH_4^+) in electrolysis products was conducted on a Bruker AVANCE III HD 600 MHz NMR spectrometer.

Quantification of reaction products.

UV–Vis spectrophotometry and ^{1}H NMR spectroscopy were used to analyze the liquid products, while gaseous products generated during electrolysis were qualitatively and quantitatively analyzed using a GC 9790plus gas chromatograph (Fuli Instruments, Zhejiang, China). The specific detection methods are detailed as follows:

1. Determination of urea

Urea was determined by two methods. Diacetyl monoxime method: Solution A was

prepared by mixing 50 mL concentrated H_3PO_4, 150 mL concentrated H_2SO_4, and 300 mL deionized water, followed by adding 50 mg $FeCl_3$. Solution B was prepared by dissolving 2.5 g diacetyl monoxime and 50 mg thiosemicarbazide in 500 mL deionized water. 1 mL sample was mixed with 2 mL Solution A and 1 mL Solution B, heated at 100 °C for 15 min, and cooled to room temperature. Absorbance spectra were recorded from 450 to 650 nm using a UV–Vis spectrophotometer. Calibration curves were established using standard urea solutions (0, 0.5, 1, 2, 3, 5 $\mu g \cdot mL^{-1}$), and linear fitting was performed at 525 nm in **Figure S**3.

Urea yield was calculated as:

$$Yield_{\mathrm{urea}} = \frac{CV}{tm}$$

where C = urea concentration determined from the calibration curve ($\mu g \cdot mL^{-1}$), V = total volume of catholyte (mL), t = electrolysis time (h), m = mass of loaded catalyst (mg).

Urease method: The 50 g L^{-1} disodium EDTA solution was diluted to 10 g L^{-1} as a buffer solution. Urease solution (5 mg mL^{-1}) was prepared using this buffer. 1.8 mL electrolyte was mixed with 0.2 mL urease solution and incubated at 40 °C for 40 min to fully hydrolyze urea. After cooling to room temperature, the total ammonia concentration (C_2, including original NH_3 and NH_3 from urea decomposition) was determined by the indophenol blue method. The initial ammonia concentration (C_1) was measured by mixing 1.8 mL electrolyte with 0.2 mL buffer solution, followed by the same indophenol blue procedure.

The urea concentration was calculated as:

$$\mathrm{C}_{\mathrm{urea}} = \frac{(C_2\text{-}C_1)\times 60.6}{2\times 17}$$

Faradaic efficiency (FE) was calculated as:

$$\mathrm{FE} = \frac{16FCV}{60.06\times Q}\times 100\%$$

where 16 = number of transferred electrons, F = Faraday constant (96485.3 $C \cdot mol^{-1}$), C = urea concentration, V = total volume of catholyte, 60.06 = molar mass of urea, Q = total charge passed during electrolysis.

2. Determination of ammonia

Indophenol blue method

Solution A was prepared by dissolving 20 g NaOH, 25 g salicylic acid, and 25 g sodium citrate in deionized water and diluting to 500 mL. Solution B was 0.05 M NaClO solution. Solution C was prepared by dissolving 5 g sodium nitroprusside in deionized water and diluting to 500 mL.

A total of 2 mL of sample was mixed with 2 mL Solution A, 1 mL Solution B, and 0.2 mL Solution C. The mixture was kept in the dark for 2 h and then left standing for 2 h. The absorbance at 655 nm was measured by UV–Vis spectroscopy.

A stock solution of NH_4Cl was prepared in the 0.1 M $KHCO_3$ + 50 mM KNO_3 electrolyte. A series of standard ammonia solutions (0, 0.25, 0.5, 1.0, 2.5, 5 $\mu g \cdot mL^{-1}$) were used to establish the calibration curve by plotting absorbance at 655 nm against concentration in **Figure S**4.

The yield of ammonia was calculated as:

$$Yield_{\mathrm{NH3}} = \frac{CV}{tm}$$

where C is the NH_3 concentration determined from the calibration curve ($\mu g \cdot mL^{-1}$), V is the total volume of electrolyte (mL), t is the electrolysis time (h), and m is the mass of loaded catalyst (mg).

Faradaic efficiency was calculated as:

$$\mathrm{FE} = \frac{8FCV}{17Q}$$

where 8 is the number of transferred electrons from NO_3^- to NH_3, F is the Faraday constant (96485.3 $C \cdot mol^{-1}$), C is the concentration of NH_3 in electrolyte, V is the total volume of catholyte, 17 is the molar mass of NH_3, and Q is the total charge passed during electrolysis.

^1H NMR qualitative analysis of ammonia

To verify the nitrogen source, control electrolyses were performed using ^{14}N- and ^{15}N-labeled nitrate as nitrogen sources. For sample preparation: 500 μL of catholyte

was collected after electrolysis and acidified with 10 μL of 1 M HCl to convert ammonia into NH_4^+. Then 50 μL of DMSO-d_6 was added and mixed thoroughly before transfer into an NMR tube for ^{1}H NMR measurement. Water suppression was employed to reduce solvent interference, and 64 scans were accumulated to ensure a sufficient signal-to-noise ratio. The characteristic signal of NH_4^+ appeared at approximately 6.8 ppm, which was used to confirm the formation of ammonia. Combined with $^{14}N/^{15}N$ isotopic comparison, the nitrogen source of ammonia was further identified.

3. Determination of nitrite

Griess method

Griess reagent was freshly prepared by dissolving 4 g sulfanilamide and 0.2 g N-(1-naphthyl)ethylenediamine dihydrochloride in 50 mL deionized water, followed by slowly adding 10 mL phosphoric acid. The electrolyte was diluted appropriately. 0.1 mL Griess reagent was added to 5 mL diluted electrolyte, mixed well, and incubated for 20 min. Absorption spectra were recorded from 425 to 650 nm using a UV–Vis spectrophotometer.

A stock solution of KNO_2 was prepared in the 0.1 M $KHCO_3$ + 50 mM KNO_3 electrolyte. A series of standard NO_2^- solutions (0, 0.5, 1.5, 2, 3 μg·mL^{-1}) were used for calibration. The calibration curve was established by linear fitting of absorbance at 540 nm versus concentration in **Figure S**5.

The yield and Faradaic efficiency of NO_2^- were calculated as:

$$Yield_{\mathrm{NO_2^-}} = \frac{CV}{tm}$$

$$\mathrm{FE} = \frac{2FCV}{46Q} \times 100\%$$

where C = NO_2^- concentration from calibration curve (μg·mL^{-1}), V = total electrolyte volume (mL), t = electrolysis time (h), m = mass of loaded catalyst (mg), 2 = number of transferred electrons from NO_3^- to NO_2^-, F = Faraday constant (96485.3 C·mol^{-1}), 46 = molar mass of NO_2^-, Q = total charge passed during electrolysis.

4. Detection and quantification of gaseous products

Gaseous products generated during electrolysis were identified and quantified by gas chromatography. The outlet gas from the cathodic chamber was directly injected into the gas chromatograph equipped with a thermal conductivity detector (TCD) and a flame ionization detector (FID) coupled with a methanizer. TCD was used for the detection of permanent gases such as H_2, while FID was employed for the quantification of carbon-containing gases such as CO. To ensure representative and reproducible sampling, gaseous products were collected after steady-state potentiostatic electrolysis, with the gas flow rate and sampling duration recorded.

The Faradaic efficiencies for H_2 and CO were calculated as follows:

$$\mathrm{FE} = \frac{2FS_1CPV}{S_2IRT} \times 100\%$$

where 2 = number of transferred electrons for the formation of H_2 and CO, F = Faraday constant (96485.3 C·mol^{-1}), S_1 = peak area of product, S_2 = peak area of standard gas, C = concentration of standard gas, I = current density during gas collection, v = CO_2 flow rate (30 mL·min^{-1}), R = gas constant (8.314 J·mol^{-1}·K^{-1}), T = temperature (298.15 K), P = pressure (101325 Pa).

5. Theoretical calculations

The calculations were obtained via the Vienna ab initio simulation program. For the treatment of electronic exchange and correlation effects, the Perdew-Burke-Ernzerhof (PBE) functional, rooted in the generalized gradient approximation (GGA), was employed. The cut-off energy of the plane-wave basis set was 450 eV for energy convergence. The Brillouin zone was sampled with a 2 × 2 × 1 k-point to achieve sufficient precision. A vacuum region of 15 Å was created at the top of the model to minimize spurious interactions between periodic cells. The convergence threshold for electronic self-consistency was 10−5 eV. The convergence threshold for ionic relaxation was set at 0.03 eV/Å.

Free energy change (ΔG) in each adsorbed intermediate was computed using the

following expression:

$$\Delta G = \Delta E + \Delta EZPE - TS$$

where ΔE stands for changes in electron energy, $\Delta EZPE$ represents zero-point energy, and S for the entropy associated with the adsorption of intermediates. The thermodynamic corrections at the reaction temperature (298 K) were calculated using the VASPKIT 1.4.1 software suite.

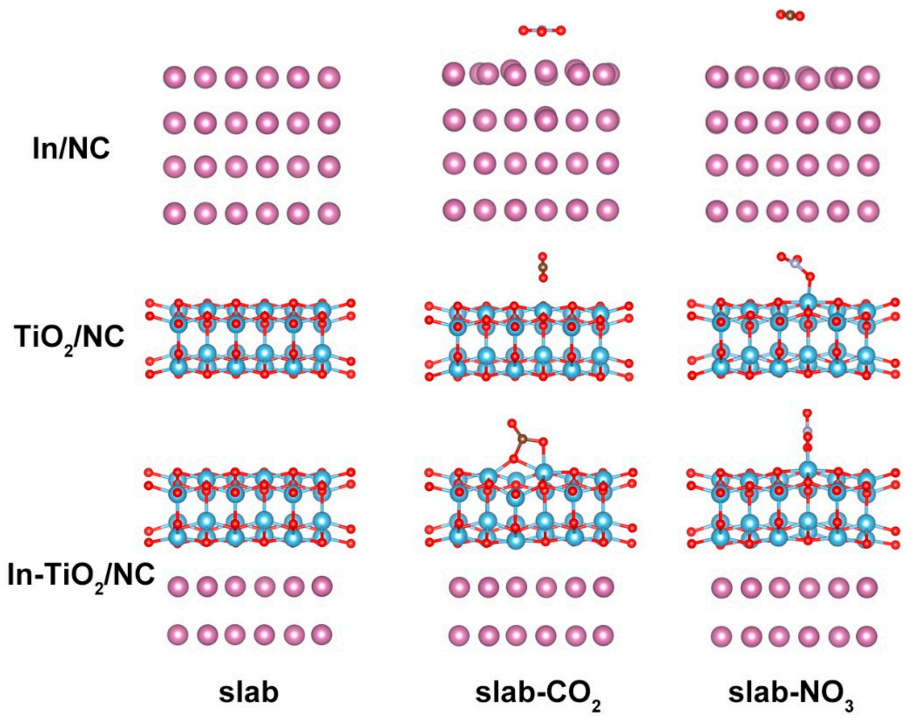

Figure S1. Optimized slab models of In/NC, TiO_2/NC, and In–TiO_2/NC before and after CO_2 or NO_3^- adsorption.

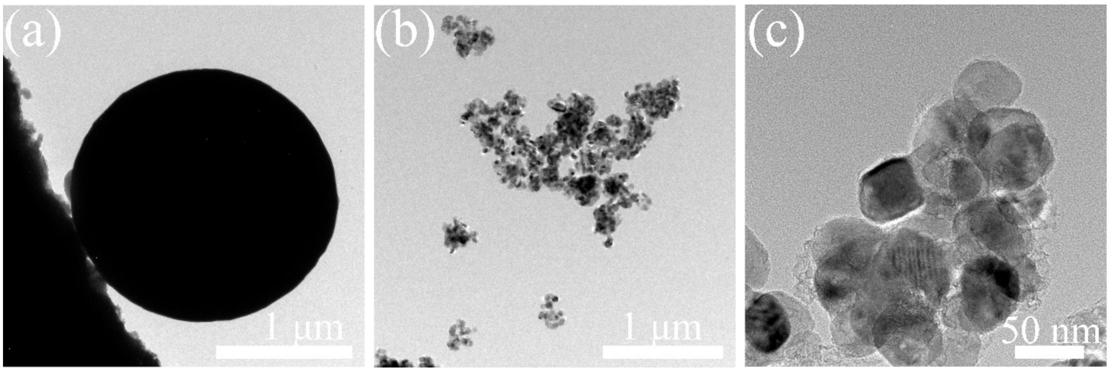

Figure S2. TEM images of (a) In/NC and (b,c) TiO_2/NC at different magnifications.

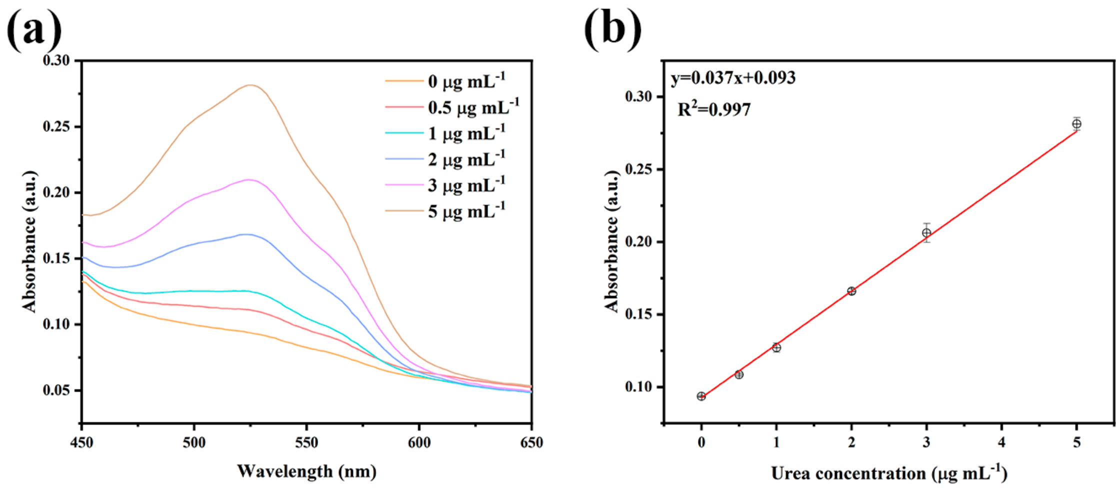

Figure S3. (a) Absorbance curves of Urea at different concentrations; (b) standard curve of Urea.

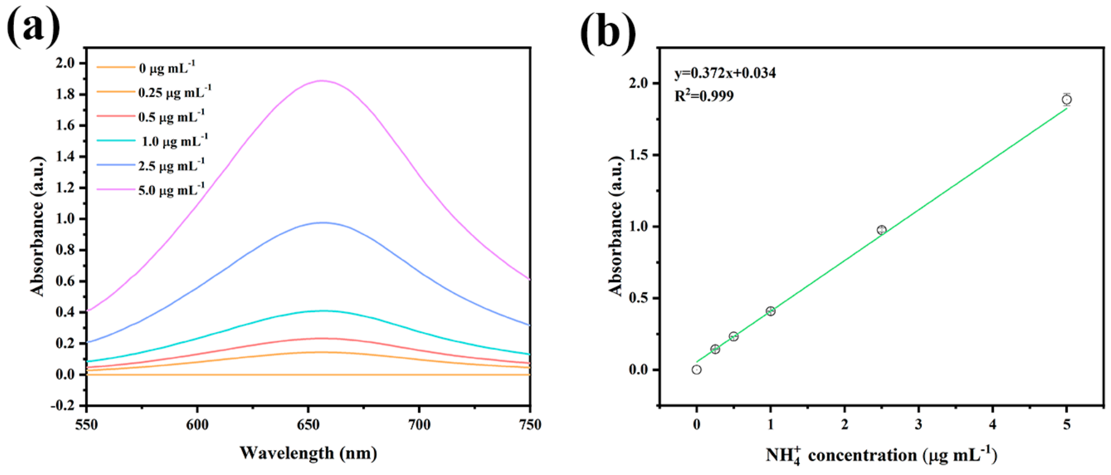

Figure S4. (a) Absorbance curves of NH_4^+ at different concentrations; (b) standard curve of NH_4^+.

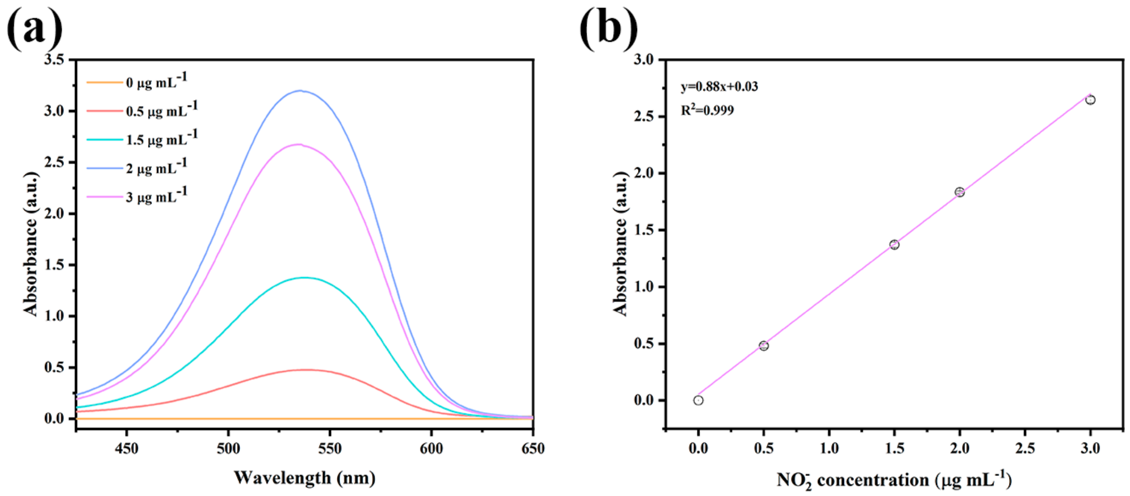

Figure S5. (a) Absorbance curves of NO_2^- at different concentrations; (b) standard curve of NO_2^-.

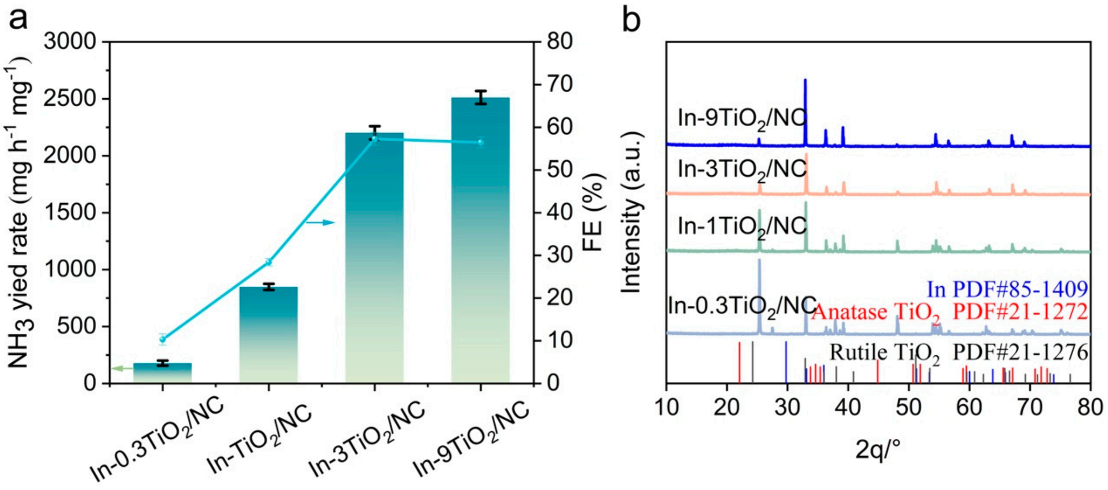

Figure S6. (a) Ammonia nitrogen performance of In–TiO_2/NC with different molar amounts of TiO_2 introduced and (b) XRD patterns.

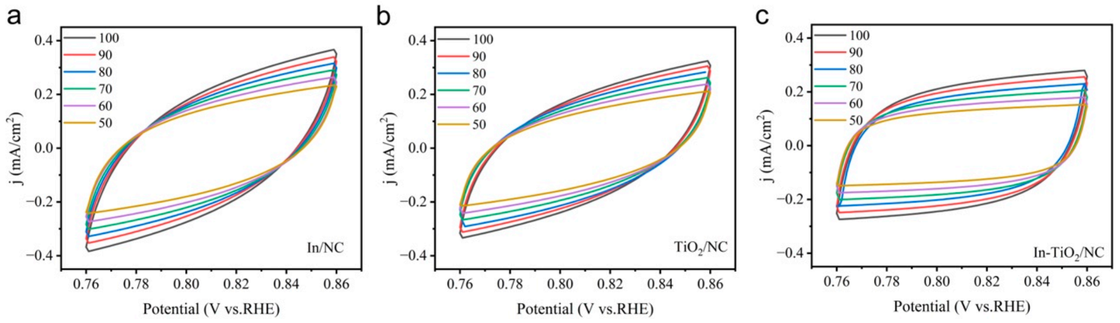

Figure S7. CV curves of (a) In/NC; (b) TiO_2/NC; (c) In–TiO_2/NC recorded at different scan rates.

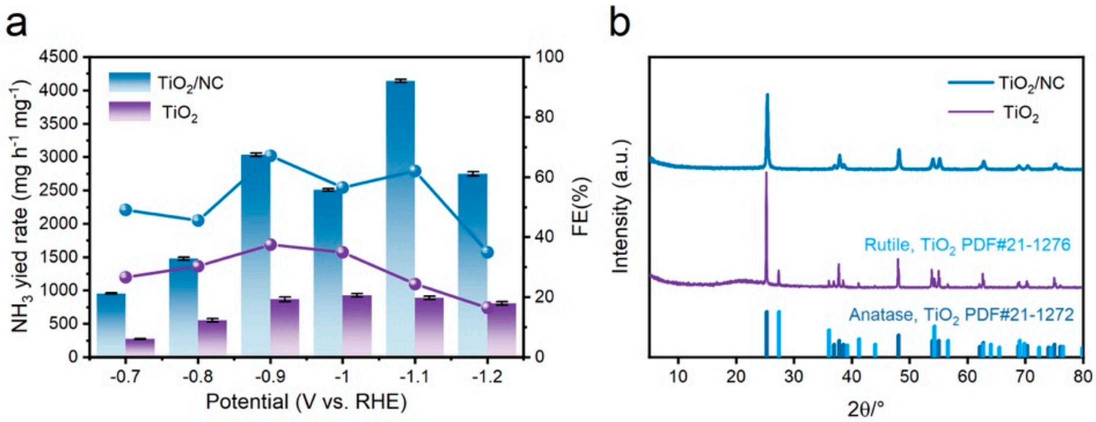

Figure S8. Electrocatalytic performance of TiO_2/NC and pure TiO_2 at different potentials for (a) ammonia synthesis; (c) corresponding XRD patterns.

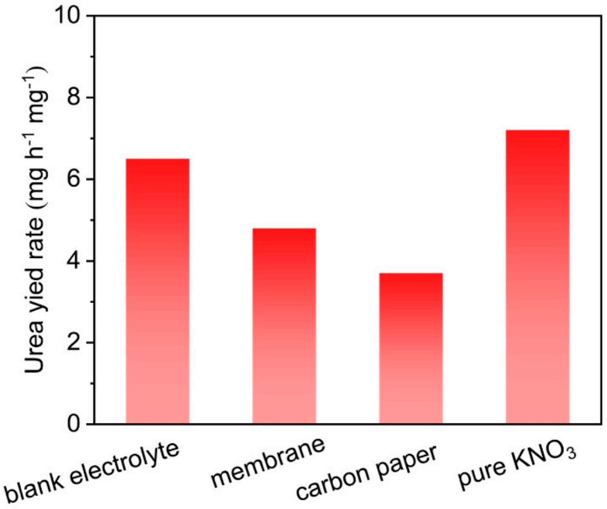

Figure S9. Urea concentrations detected in component-specific blank systems containing the electrolyte, membrane, carbon paper, or pure KNO_3 under the same electrochemical and analytical conditions. No catalyst was added.

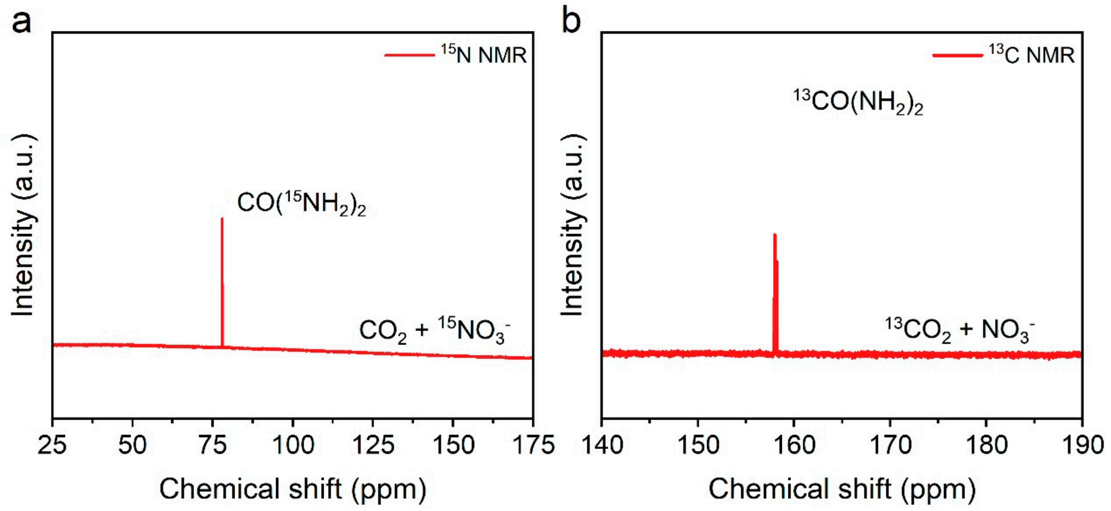

Figure S10. Isotope-labeling NMR spectra of urea: (a) ^{15}N NMR spectrum from the $CO_2/{}^{15}NO_3^-$ co-reduction experiment; (b) ^{13}C NMR spectrum from the $^{13}CO_2/NO_3^-$ co-reduction experiment.

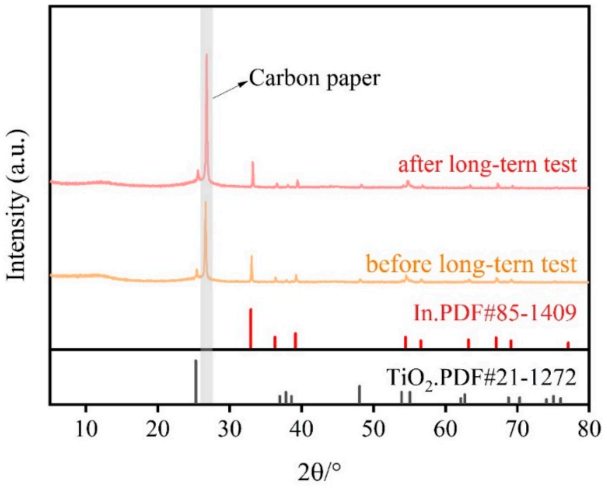

Figure S11. XRD patterns of In–TiO_2/NC before and after the 30 h stability test at −1.0 V vs. RHE.

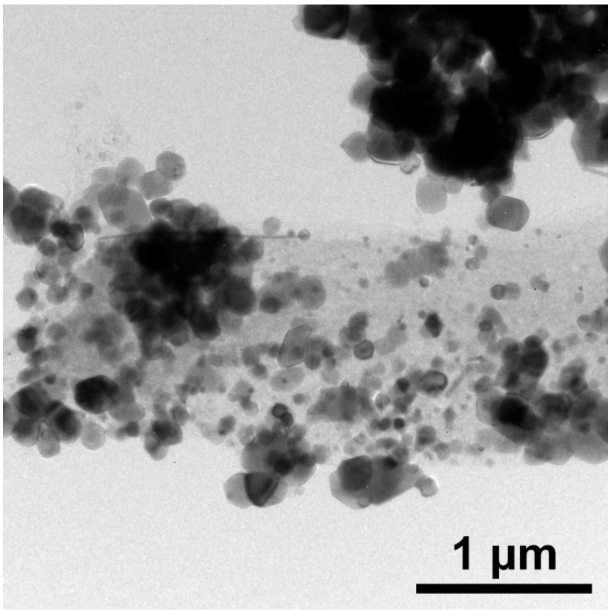

Figure S12. TEM image of In–TiO_2/NC after the 30 h chronoamperometric stability test at −1.0 V vs. RHE.

Table S1. Comparison of electrocatalytic urea synthesis performance of reported bimetallic and defect-engineered catalysts.

Catalyst	Electrolyte	Potential V vs. RHE	FE_{Urea} (%)	Urea yield((mmol h^{-1} g_{cat}^{-1})	Ref.
Fe SAC	0.1 M $KHCO_3$ + 50 mM KNO_3	-1.5	8.92	2.03	1
Fe-Ni-DASC	0.1 M $KHCO_3$ + 50 mM KNO_3	-1.5	17.8	20.2	1
B-FeNi-DASC	0.05 M KNO_3	-1.5	17.8	20.2	1
Te-Pd NCs	0.1 M $KHCO_3$ + 0.01 M KNO_2	-1.1	12.20	6.08	2
Cu-TiO_2	0.2 M $KHCO_3$ + 0.02 M KNO_2	-0.4	27.30	2.96	3
ZnO-V	$NaHCO_3$ + $NaNO_2$	-0.79	21.26	8.28	4
Cu_2O@ZnO	0.2 M $KHCO_3$ + 50 mM KNO_3	-1.02	9.28	3.65	5
$BiFeO_3$/$BiVO_4$	0.1 M $KHCO_3$	-0.4	17.18	2.47	6
CuPc-NTs	0.1 M $KHCO_3$	-0.6	12.99	2.39	7
$CoRuN_6$	0.1 M KNO_3	-0.6	25.31	8.98	8
Cu_1–CeO_2	0.05 M KNO_3	-1.6	5.4	52.84	9
In-TiO_2/NC	0.1 M $KHCO_3$ + 50 mM KNO_3	-1.0	11.7	10.3(618.9 μg h^{-1} mg_{cat}^{-1})	This work

Table S2. The atomic ratios of In–TiO_2/NC before and after the electrolysis from the EDX results.

	In	Ti	N	C	O
Initial	1.68	26.61	1.59	53.19	16.93
After the electrolysis	1.57	27.24	1.61	53.69	15.89

[1] Zhang, X.; Zhu, X.; Bo, S.; et al. Identifying and tailoring C–N coupling site for efficient urea synthesis over diatomic Fe–Ni catalyst. *Nat. Commun.* **2022**, *13*, 5337. https://doi.org/10.1038/s41467-022-33066-6

[2] Feng, Y.; Yang, H.; Zhang, Y.; Huang, X.; Li, L.; Cheng, T.; Shao, Q. Te-doped Pd nanocrystal for electrochemical urea production by efficiently coupling carbon dioxide reduction with nitrite reduction. *Nano Lett.* **2020**, *20*, 8282–8289. https://doi.org/10.1021/acs.nanolett.0c03400

[3] Cao, N.; Quan, Y.; Guan, A.; Yang, C.; Ji, Y.; Zhang, L.; Zheng, G. Oxygen vacancies enhanced cooperative electrocatalytic reduction of carbon dioxide and nitrite ions to urea. J. Colloid Interface Sci. 2020, 577, 109–114. https://doi.org/10.1016/j.jcis.2020.05.014

[4] Meng, N.; Huang, Y.; Liu, Y.; Yu, Y.; Zhang, B. Electrosynthesis of urea from nitrite and CO_2 over oxygen vacancy-rich ZnO porous nanosheets. Cell Rep. Phys. Sci. 2021, 2, 100378. https://doi.org/10.1016/j.xcrp.2021.100378

[5] Meng, N.; Ma, X.; Wang, C.; Wang, Y.; Yang, R.; Shao, J.; Huang, Y.; Xu, Y.; Zhang, B.; Yu, Y. Oxide-derived core–shell Cu@Zn nanowires for urea electrosynthesis from carbon dioxide and nitrate in water. ACS Nano 2022, 16, 9095–9104. https://doi.org/10.1021/acsnano.2c01177

[6] Yuan, M.; Chen, J.; Bai, Y.; Liu, Z.; Zhang, J.; Zhao, T.; Shi, Q.; Li, S.; Wang, X.; Zhang, G. Electrochemical C–N coupling with perovskite hybrids toward efficient urea synthesis. Chem. Sci. 2021, 12, 6048–6058. https://doi.org/10.1039/D1SC01467F

[7] Mukherjee, J.; Paul, S.; Adalder, A.; et al. Understanding the site-selective electrocatalytic co-reduction mechanism for green urea synthesis using copper phthalocyanine nanotubes. *Adv. Funct. Mater.* **2022**, *32*, 2200882. https://doi.org/10.1002/adfm.202200882

[8] Liu, C.; Tong, H.; Wang, P.; et al. The asymmetric orbital hybridization in single-atom dimers for urea synthesis by optimizing the C–N coupling reaction pathway. *Appl. Catal. B Environ.* **2023**, *336*, 122917. https://doi.org/10.1016/j.apcatb.2023.122917

[9] Wei, X.; Liu, Y.; Zhu, X.; Bo, S.; Xiao, L.; Chen, C.; et al. Dynamic reconstitution between copper single atoms and clusters for electrocatalytic urea synthesis. *Adv. Mater.* **2023**, *35*, e2300020. https://doi.org/10.1002/adma.202300020
